# Editorial: The noneconomic and economic wellbeing of immigrants

**DOI:** 10.3389/fsoc.2022.1009743

**Published:** 2022-08-31

**Authors:** Dina Maskileyson, Debora Pricila Birgier

**Affiliations:** ^1^Faculty of Management, Economics and Social Sciences, The Institute of Sociology and Social Psychology, University of Cologne, Cologne, Germany; ^2^Unit for Economic History, Department of Economy and Society, School of Business, Economics and Law, University of Gothenburg, Gothenburg, Sweden; ^3^Department of Economic History, Center for Economic Demography, School of Economics and Management, Lund University, Lund, Sweden

**Keywords:** economic wellbeing, noneconomic wellbeing, immigration, life satisfaction and happiness, self-perceived health, social integration

Questions relating to immigrant integration are centrally positioned in the migration literature. Scholars of immigration focus on two main dimensions of wellbeing in order to assess the integration of immigrants into the host society: economic (i.e., labor force participation, occupational attainment, and earnings) and noneconomic (i.e., physical and mental health, subjective wellbeing, life satisfaction). While researchers have long addressed these dimensions, a number of important gaps in the evidence remain. For instance, the long-term consequences of immigration for immigrants' economic and noneconomic wellbeing are still insufficiently addressed. Therefore, this thematic issue aims to explore the consequences of migration for noneconomic and economic wellbeing. That is, this thematic issue presents different studies focusing on economic and health outcomes as well as subjective wellbeing and life satisfaction of different immigrant' groups and across different countries and contexts.

In terms of economic incorporation, it has been established that upon arrival at a host country, most immigrant groups must contend with lower incomes and employment rates in their new labor markets. The lower earnings of immigrants are attributed to skill disparities, lack of language proficiency, information gaps, and discrimination. However, as immigrants spend more time in the host society, labor market outcomes tend to converge toward the levels enjoyed by natives. The long-term consequences of immigration for immigrants' economic wellbeing and the distribution of economic resources across different immigrant groups are yet insufficiently addressed in the literature.

In terms of noneconomic wellbeing, studies report that while immigrants tend to be less satisfied with their lives in their destination countries relative to natives, in many cases, they still report higher life satisfaction than their counterparts in their country of origin. Most of the research on the health subject reports that immigrants arrive healthier, but their health deteriorates with increasing length of stay and generational status in the destination country. This phenomenon is addressed in the literature as the healthy immigrant effect. Upon arrival in the host country, immigrants tend to have comparatively better health profiles (in terms of mortality rate, chronic conditions, and mental health) than the native-born population, despite their lower socioeconomic status, experiences of discrimination, and reduced access to healthcare systems. However, over time and change in generational status, this health advantage frequently deteriorates, despite the relative improvement in socioeconomic status. The reasons underlying the healthy immigrant effect remain a topic for vigorous research and debate.

This thematic issue covers studies that analyze in a theory-driven way and use survey data on such various aspects of immigrants' experience in the host society as life satisfaction and happiness (Arat and Bilgili; Brockmann; Ambrosetti et al.; Shen and Kogan et al.), parental support (Mangrio et al.), self-perceived health (Semyonov-Tal and Maskileyson; Ambrosetti et al.), social integration and national identification (Becker), and motherhood penalty in the labor market (Achouche). The studies analyze these questions from a comparative or longitudinal perspective.

The first paper, “*Transnational and Local Co-ethnic Social Ties as Coping Mechanisms Against Perceived Discrimination - A Study on the Life Satisfaction of Turkish and Moroccan Minorities in the Netherlands*”, authored by Arat and Bilgili, examines whether and to what extent minorities' local and transnational co-ethnic social ties mitigate the negative effects of perceived discrimination on life satisfaction. The authors focus on the experiences of Turkish and Moroccan minorities and discuss whether co-ethnic social ties, both locally and transnationally embedded, can be considered as coping mechanisms against perceived discrimination. In addition, they investigate whether these mechanisms work differently for first- and second-generation minorities. Using Netherlands Longitudinal Life-course Study, Arat and Bilgili reveal that perceived discrimination is positively associated with local co-ethnic social ties in the Netherlands, which consequently predicts higher life satisfaction for both generations. They also demonstrate that discrimination is associated with stronger transnational co-ethnic social ties only among the second-generation, but not the first generation. The authors conclude, however, that having transnational ties is beneficial for the life satisfaction of both generations. Thus, they highlight the importance of recognizing transnational embeddedness of minorities and studying the effects of transnational co-ethnic social ties on subjective wellbeing outcomes, especially for second-generation minorities.

The second paper, “*The Need for Parental Support for Migrant Parents in Transition Into Sweden: A Perspective*”, authored by Mangrio et al., discusses how the Swedish Child Health Services (CHS) can support newly arrived immigrant families and address the need for improvement in the parental support offered to migrant parents during the transition into the Swedish society. This study focuses on the advantages of using a community-based participatory research approach together with the Swedish CHS to identify and apply culturally appropriate support programs to increase health literacy among migrant parents. The authors suggest that healthcare professionals in Sweden should aim on taking an inclusive approach to provide parental support to migrant parents, where migrant parents themselves are actively involved in the development of support programs. Mangrio et al. argue that this approach will provide migrant families with knowledge and support based on their needs and challenges.

The third paper, “*Unhealthy Immigrants: Sources for Health Gaps Between Immigrants and Natives in Israel*” by Semyonov-Tal and Maskileyson, focuses on sources for health disparities between immigrants and native-born in the context of the “returning diaspora” model of Israeli society. The authors distinguish between three major origin groups of immigrants: the former Soviet Union, Western Europeans or the Americans (mostly Ashkenazim), and Asians or North Africans (mostly Sephardim). Using data from the Israeli National Health Interview Survey (2013–2015), Semyonov-Tal and Maskileyson provide a decomposition analysis of the illness gaps between native-born and subgroups of immigrants. Their findings reveal that the health status of all immigrant groups is poorer than that of native-born Israelis. The nativity–illness gap is most pronounced in the case of male immigrants (from Europe or the Americas or South Africa or Australia) and for female immigrants (from countries in the Middle East or North Africa) and least pronounced in the case of immigrants arriving from the former Soviet Union for both gender groups. Decomposition of the gaps into components reveals that some portion of the illness gap can be attributed to nativity status, but the largest portion of the gap is attributed to demographic characteristics. Neither socioeconomic status nor health-related behavior accounts for a substantial portion of the nativity–illness gap for all subgroups of immigrants.

The fourth study, “*Why Are Newcomers So Happy? Subjective Well-being of First-Generation Immigrants in Germany*”, authored by Brockmann, tests if personality selectivity, purposive adaptation, and social resilience separately or in tandem explain why subjective wellbeing of newcomers remains high even in times of objective disadvantage. Using German panel data (GSOEP) from 5,008 first-generation immigrants for the years 1984–2014 and official data, growth curve models show that newcomers are a selected group concerning their open and less neurotic personalities and that these personal characteristics are distinctly associated with happiness. Also, newcomers immediately compare their income to the standards in the host society but not their family life. This contributes to boosting their subjective wellbeing as well. For more than 30 years, first-generation immigrants have used their country of origin as a reference point, thus protecting the positive association of intimate relationships and happiness. Finally, newcomers are highly capable of recovering from social loss. Brockmann argues that the economic integration of newcomers should be fast and easy, while family reunification and integration should follow only with a time lag.

The fifth study, “*The Impact of Pre- and Postarrival Mechanisms on Self-rated Health and Life Satisfaction Among Refugees in Germany*”, authored by Ambrosetti et al., examines the evolution of refugees' wellbeing in the first years after their arrival in Germany. In contrast to other immigrants (e.g., labor migrants), refugees experience higher risks of unexpected and traumatic events and insecurity before and during their migration and face various legal and structural barriers in the receiving country. This study contributes to the existing literature by exploring the possible pre- and post-arrival determinants of refugees' life satisfaction and self-rated health upon arrival in Germany and their development over time in the process of becoming established. Applying linear regression and panel models with recent longitudinal data from the IAB-BAMF-SOEP Survey of Refugees in Germany, Ambrosetti et al. find significant effects of prearrival factors, such as traumatic experiences and the complexity of migration, on both life satisfaction and self-rated health at the time of the first interview. Regarding postarrival factors, their results suggest that improvement in language proficiency and labor market status significantly shape refugees' life satisfaction and self-rated health. The time-dynamic analyses reveal substantial improvements in life satisfaction upon the approval of refugee status and the transition from shared housing to private accommodations. However, the authors find no improvements in self-rated health due to legal status but rather deterioration effects due to long-term residence in shared housing.

The sixth paper, “*Gendered Body Mass and Life Satisfaction Among Youth in Three Western European Immigrant-Receiving Countries*”, by Shen and Kogan, demonstrates distinctive patterns of the association between body weight and life satisfaction for adolescent boys and girls. They examine such patterns by bringing multiple mediating factors into one theoretical framework centered on normative perceptions. By drawing data from the first wave of the CILS4EU that captures 14–15-year-olds in Germany, the Netherlands, and Sweden, findings show that psychological factors, indicated by self-esteem and mental state, explain the association between BMI and life dissatisfaction substantially for both boys and girls. Relationships with parents (particularly among boys) and relationships with peers (particularly among girls) also play significant roles. Interestingly, the association between being underweight and life satisfaction varies across ethno-racial groups among girls but not among boys. Girls originating from Eastern Europe tend to gain more life satisfaction when being underweight, whereas girls rooted in Sub-Saharan African and Caribbean countries display consistently low levels of life satisfaction when being underweight.

The sevenths paper, “*Migrants' Social Integration and Its Relevance for National Identification: An Empirical Comparison Across Three Social Spheres*”, authored by Becker, analyzes and compares the relationship between different forms of social integration and national identification of first- and second-generation migrants in Germany. Becker analyzed data from a 2013 cooperation between the Institute for Employment Research (IAB) and the German Socio-Economic Panel (SOEP), that is, the IAB-SOEP Migration Sample, as well as the 2014 wave of the SOEP. The subsample used included 2,780 first- and second-generation migrants living in Germany. The results indicate that not all kinds of contact are equally linked to national identification. In neither the cross-sectional models nor the lagged models living together with native family members significantly linked to national identification. Similarly, the association between having predominantly native co-workers and national identification was insignificant when controlling for migrant-specific characteristics. Only the relation with having predominantly native friends was significant and positive across all models. The author concluded that when it comes to migrants' national identification, native friends might be the most relevant form of contact with natives.

Finally, the eighth paper, “*The Motherhood Penalty of Immigrants in France: Comparing the Motherhood Wage Penalty of Immigrants from Europe, the Maghreb, and Sub-Sahara with Native-Born French Women*”, authored by Achouche, examines whether the negative effect of motherhood on wages is higher for immigrants than it is for the native population; and how this effect may vary across different immigrant regions of origin. A series of linear regression models were calculated using data from the Enquête Revenus Fiscaux et Sociaux from 2009 to 2012 (INSEE, 2009–2012) to address these questions. The results revealed substantial differences in the motherhood penalty between the different regions of origin and asserted the existence of an especially pronounced motherhood penalty for mothers from the Maghreb. Given the gap in the research regarding the cost of motherhood for immigrants in the host country's labor market, this research sheds light on specific mechanisms influencing the integration patterns of immigrant women. Moreover, by choosing France, which is one of the main immigration destinations in Europe, and a country where the motherhood penalty for the native population is almost non-existent, this study provides a new perspective on the intersection of motherhood, immigration, and region of origin in the immigrants' labor-market integration process.

## Author contributions

All authors listed have made a substantial, direct, and intellectual contribution to the work and approved it for publication.

## Conflict of interest

The authors declare that the research was conducted in the absence of any commercial or financial relationships that could be construed as a potential conflict of interest.

## Publisher's note

All claims expressed in this article are solely those of the authors and do not necessarily represent those of their affiliated organizations, or those of the publisher, the editors and the reviewers. Any product that may be evaluated in this article, or claim that may be made by its manufacturer, is not guaranteed or endorsed by the publisher.

